# Cesarean Scar Ectopic Pregnancy: The Lurking Danger in Post Cesarean Failed Medical Abortion 

**Published:** 2019-12

**Authors:** Monika Anant, Anita Paswan, Chandra Jyoti

**Affiliations:** Department of Obstetrics and Gynecology, AIIMS Patna, Patna, India

**Keywords:** Ectopic Pregnancy, Scar Ectopic, Laparoscopy, Methotrexate

## Abstract

Cesarean scar pregnancy (CSP) is a potentially life threatening ectopic pregnancy where a missed diagnosis is commoner than an accurate diagnosis. Incidence of Ectopic pregnancy is 1 – 2 % and cesarean scar ectopic occurs in about (0.05%) 1 in 2000 of all pregnancies. With increasing cesarean section rates worldwide, CSP is bound to increase with its dreaded complications like uterine rupture and catastrophic hemorrhage. Three patients misdiagnosed as incomplete miscarriages in post cesarean pregnancies in other centers were found to be CSP in Gynaecology department of a tertiary level hospital. All three patients were managed successfully, two surgically and one medically.

## Introduction

Cesarean scar pregnancy (CSP) is a potentially life-threatening ectopic pregnancy where a missed diagnosis is commoner than an accurate diagnosis. Incidence of ectopic pregnancy is 1 – 2 % and cesarean scar ectopic occurs in about (0.05%) 1 in 2000 of all pregnancies (1, 2). With increasing cesarean section rates worldwide, incidence of CSP is bound to increase with its dreaded complications like uterine rupture and catastrophic hemorrhage. 

The probable mechanism of implantation at the scar maybe a lower segment myometrial wedge defect or a minute fistula in the scar so that the sac is surrounded on all sides by the myometrium (3). Apart from cesarean sections, manual removal of placenta, previous dilatation curettage or even an in-vitro fertilized pregnancy may cause scar implantations, also called myometrial pregnancies but are even rarer (4).

CSP is not easy to diagnose as the presentation is too variable and it can be easily confused with intrauterine low lying gestation on ultrasound unless scrutiny of the surrounding myometrium is made. Patients may present with variable vaginal bleeding, abdominal pain or even with signs of acute rupture of uterus and hypovolemic shock as result of intraperitoneal bleed. A false-negative diagnosis may result in major complications like hysterectomy in young patient. 

With the availability of over the counter medications for abortion in India, incomplete miscarriage in first trimester is a common presentation of many patients with unwanted pregnancies. Retained products of conception (RPOC) diagnosed on ultrasound are usually managed with suction evacuation or repeated multiple doses of misoprostol. In a pregnancy with previous cesarean section, it may be hazardous to attempt suction of the RPOC, if it has implanted on the scar resulting either in massive vaginal haemorrhage or may lead to uterine perforation and intraperitoneal hemorrhage. 

Three post-cesarean patients who were misdiagnosed as incomplete miscarriages in other health centers were confirmed to be CSP in the study hospital from March 2018 - March 2019 in the Gynaecology department. Two patients were managed surgically and the third was managed medically. Informed consent has been taken form study participants for using their details and pictures.

## Case detail

**Figure 1:**
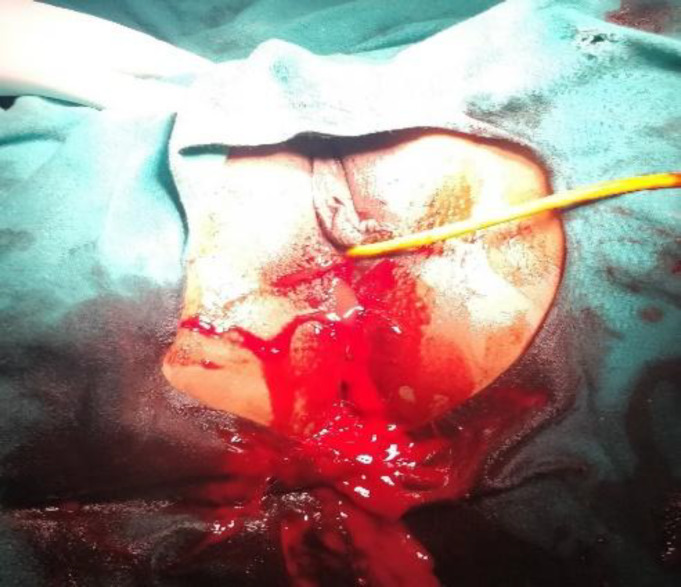
Torrential bleed on attempted suction evacuation

***Case 1:*** A 28 year, gravida 2 para 1 living 1 patient presented to the Gynaecology outpatient department with intermittent vaginal bleeding after intake of self-prescribed abortifacients 10days ago for a 6 weeks gestation. She had a cesarean section for non-progress of labor one and a half year ago. Patient was stable, ultrasound done in another center reported retained products of conception in the uterine cavity, hence a suction evacuation was planned. On attempted suction, massive vaginal bleeding of the amount of 400ml occurred when only minimal product was evacuated (Figure 1). 

**Figure 2:**
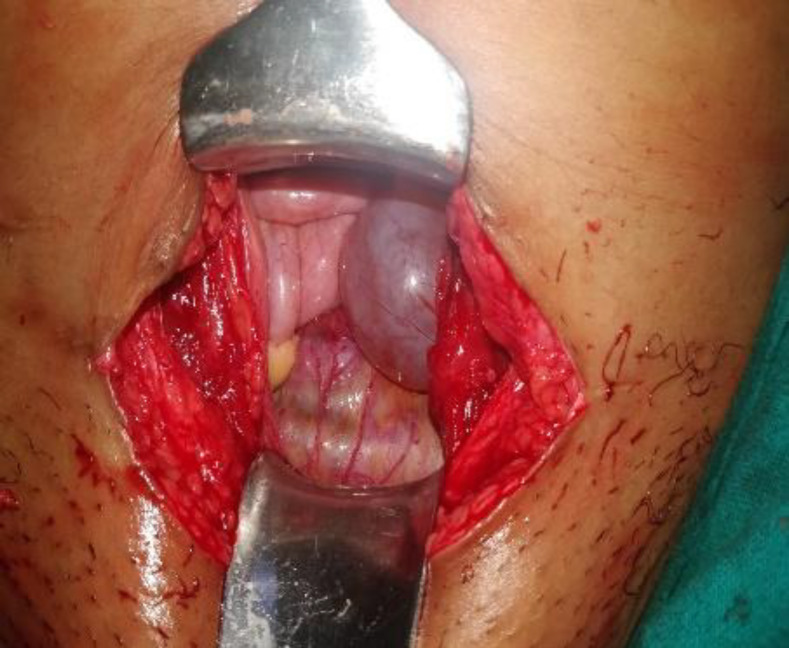
laparotomy view of CSP with thinned out bugging lower uterine segment, vascular

Attempted uterotonics, bimanual compression, cervical sutures failed to control the bleeding. Emergency laparotomy was performed and an unruptured Cesarean Scar Pregnancy was confirmed (Figure 2). The lower segment was bulging 4cm x 3cm, thinned out with multiple vessels visible but no hemoperitoneum. Bilateral tubes and ovaries were normal. The vesico-vagnal fold of peritoneum was carefully dissected and bladder pushed down following which the scar with the attached RPOC was excised. The freshened thinned out scar margin was repaired with 1-0 delayed absorbable polyglactin sutures in a single layer. 

**Figure 3:**
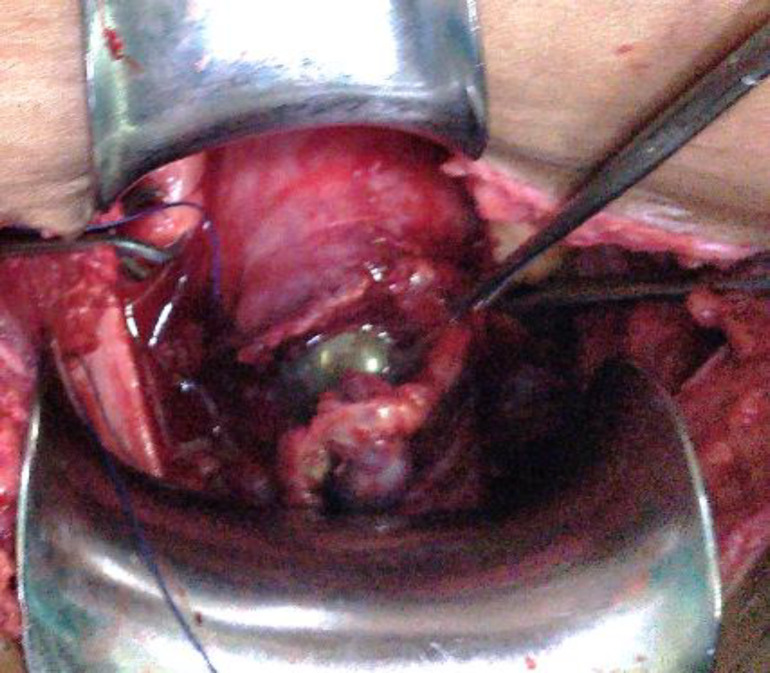
repair of scar after excision of CSP with Foley’s in utero

Intrauterine 8Fr Foley’s catheter was introduced vaginally to arrest bleeding which continued despite maximum scar excision (Figure 3). The patient received 1 unit of blood transfusion, oxytocin infusion was continued for 48 hours after which intrauterine Foley’s catheter was removed and oxytocin stopped. There were no further bleeding episodes. She was discharged after 2 days and has resumed her normal menstrual cycles.

***Case 2: ***A 26 years G3 P1 L1 A1 patient attended the emergency ward in gynaecology department, with heavy vaginal bleeding after intake of abortifacients for 7 weeks of pregnancy. Her cesarean section was done just nine months back for fetal distress. Although her vitals were stable, due to heavy bleeding on examination and opened cervical os, immediate suction evacuation was undertaken. She had an ultrasound report of intrauterine gestational sac of 7 weeks done in an outside health center in the prior week. Torrential fresh bleeding ensued following introduction of Karman’s cannula number 8. Evacuation was attempted by ovum forceps but torrential bleeding continued amounting to 700ml. Emergency laparotomy was performed and a CSP was diagnosed. The excision and repair of CSP was similar to case1 detailed above. 

This patient required 3 units of blood transfusion, had 8 days of hospital stay, febrile morbidity and was discharged on 9^th^ post-operative day in stable condition. She resumed her normal menstruation in the next cycle and was advised to use contraception regularly for 2 years. However she has presented to obstetrics recently with a 6 weeks gestation, immediate transvaginal ultrasound' has confirmed a fundal gestational sac. She has been counselled to continue pregnancy with risks explained. 

**Figure 4:**
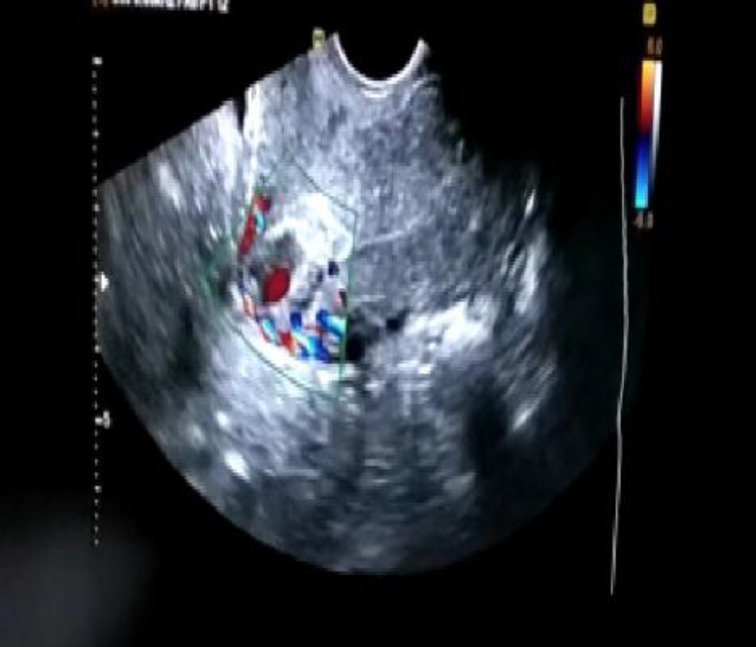
4.8 cm x2.6cm heterogeneously echogenic GS implanted at scar, increased vascularity, empty cervix and endometrial cavity

***Case 3:*** A 29 years G4P1L1A3, post cesarean (3.5yrs) presented to old with continuous bleeding per vaginum for 1 month. She had an USG of 8 weeks pregnancy 10days ago and had a dilatation evacuation done outside a week ago for termination of pregnancy. A transvaginal ultrasound with 5 MHz probe in department showed retroverted and retroflexed uterus with a 4.6 cm x 2.8 cm exophytic heterogenously echogenic gestational sac in lower uterine segment, in anteriorly thinned myometrium at the level of scar with enhanced peripheral vascularity on color and spectral Doppler with a high velocity and low impedance flow (Figure 4). 

**Figure 5:**
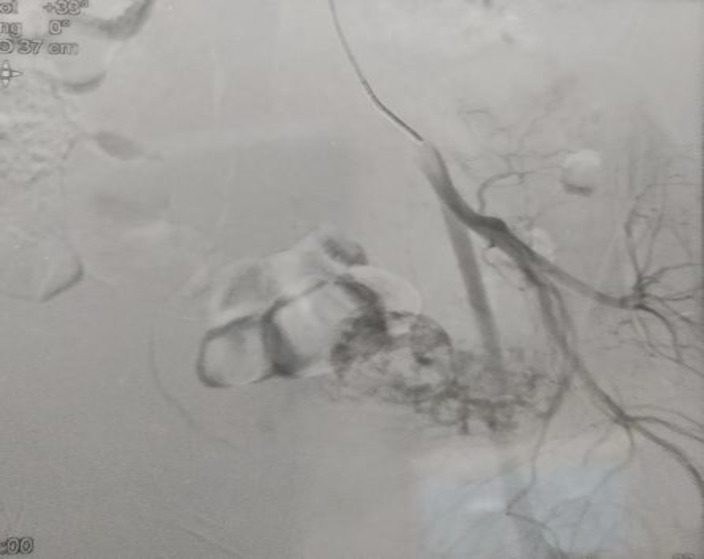
DSA shows vascular plexus formation in pelvis, with no specific AVM nidus

The cervix below and the uterine cavity above the sac were empty. There was no free fluid in pelvis and both tubes and ovaries were normal. The pregnancy test was positive, β- hCG was 1696mIU/ml. With confirmed diagnosis of unruptured CSP, medical management systemic methotrexate single dose 50 mg was given intramuscularly. Serum β- hCG values were followed weekly. The fall of β- hCG was week1: 428, week2: 404, week3: 282, week4: 112, week5: 43.2, week6: 19, week7: 4, week8: not detectable. Weekly urine pregnancy test have remained negative for 3 weeks and then monthly for 4 months. A pelvic digital subtraction angiography was performed while the patient was in follow-up which showed a hyper-vascularised pelvic structure with contrast puddles raising suspicion for aneurysm but no definite AVM nidus could be localized (Figure 5). 

**Figure 6:**
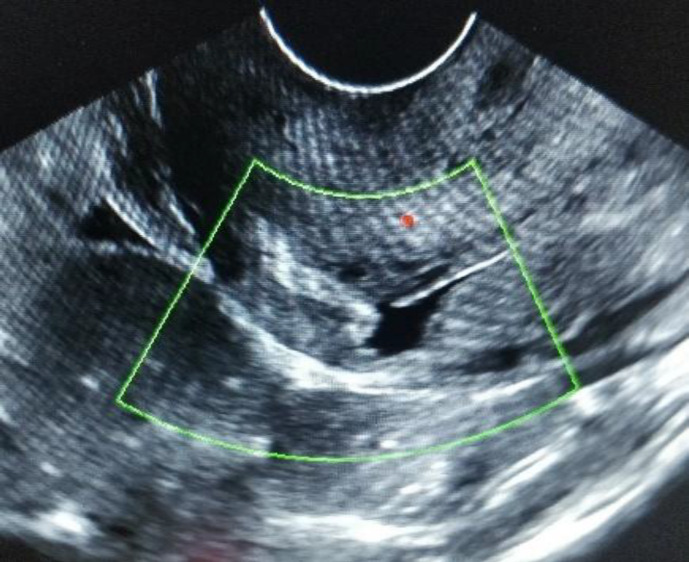
4 months post medical management by methotrexate

The resolution of the exophytic vascular mass with methotrexate therapy confirmed the diagnosis of CSP and not AVM (Figure 6).

## Discussion

Cesarean Scar Pregnancy, first described in 1978 (5), has been rising in incidence with increasing cesarean section rates around the globe. As described by Rotas et al., only 37% CSP can be incidental findings on dating scan while rest 60% can present with vaginal bleeding, bleeding with lower abdominal pain in impending rupture or hypovolemic shock with ruptured CSP (2). In stable unruptured CSP, clinical examination is usually unremarkable and not very informative, with the uterus being tender only if there is an impending rupture. So a disturbed CSP can present as emergency, while a stable CSP may present unawares to an ultrasonologist for a dating scan.

An ultrasonography preferable via the transvaginal route is the investigation of choice in diagnosing a CSP having a high diagnostic sensitivity of 86.4% (95% CI 0.763– 0.9050) (2). The proposed criteria for diagnosing CSP are:

   1) An empty uterine cavity 

   2) A clearly visible empty cervical canal 

   3) Presence of the gestation sac with or without a fetal pole with or without fetal cardiac activity in the anterior part of the uterine isthmus 

   4) Absence of or a defect in the myometrial tissue between the bladder and the sac.

   5) No adnexal mass or free fluid in pelvis should be present for diagnosing unruptured CSP (6). 

However , it becomes all the more difficult to diagnose CSP after an attempted suction or medical abortion, the diagnostic sonographic features are morphed with bleeding in cavity and cervical canal, as was seen in case 1 and 2. In case 3, a heightened awareness of CSP resulted in a correct diagnosis of CSP.

Cesarean scar pregnancy (CSP) maybe an early manifestation of abnormally invasive placenta (AIP), both having the similar histopathology. The most common ultrasound sign is a low anterior implantation of the placenta/gestational sac lying close to or in the cesraean scar (7, 8).

In some cases where sonography is inconclusive, contrast-enhanced MRI can be used as a more accurate diagnostic modality. Location of implantation, anterior myometrial thickness and bladder- uterus interface tissue can be more accurately viewed by MRI (9). 

The aim of management of CSP is to prevent uterine rupture and its associated morbidity like hysterectomy. So timely diagnosis with accurate ultrasound will allow treatment options to preserve the uterus and prevent catastrophic haemorrhage. Treatment options are individualised patient wise based on gestational age at presentation, desire for future fertility, hemodynamic status as there are no specific treatment recommendations till date. 

Expectant management should not be an option for CSP due to high risk of rupture and haemorrhage causes life threatening condition, almost one third requiring hysterectomy (10). 

Surgical treatment may be performed by laparotomy or laparoscopy to resect out the CSP and repair the uterine scar (11). Early recovery of patients are possible with resection. Transvaginal resection and suturing also has been described by Wang et al with good outcomes (12). There is a 44.1% post treatment complication rate associated with CSP and even a 5% rate of hysterectomy. Hysteroscopic resection has been reported to have quicker return of serum B hCG values and lesser requirement for follow-up than local or systemic methotrexate injection (13). 

Uterine artery embolization (UAE) is a modality of treatment but is rarely used alone. Addition of UAE reduces bleeding risk and improves success rates when combined with surgical or medical management. Dilatation and curettage alone without UAE can lead to perforation and catastrophic haemorrhage as was the seen in cases 1 and 2. However curettage under Ultrasound‐guidance has been reported to be an effective method for CSP having low risk of blood transfusion and hysterectomy (14, 15). 

Medical management is by methotrexate which is the drug of choice for all ectopic pregnancies is used in the same regimen and follow up as in other EP (16). There is a requirement of many weeks to months of follow up and there maybe risk of further rupture and surgery during the follow up period, which needs to be emphasized to the patient. Systemic methotrexate therapy is 70-80% successful (17). Local methotrexate administration or other embryocidal injection like postassium chloride can be done via transvaginal route under ultrasound guidance. Combined intramuscular and intra-gestational methotrexate injection treatment is also successful and has better success in treating CSP (17). 

In conclusion, the proper management of CSP often requires multi-modality treatment for successful outcome (18). 

To summarize the lessons from this case series , first trimester medical or surgical termination of a CSP which has been misdiagnosed as a low lying pregnancy or RPOC, increases risk of serious outcomes with heavy bleeding , need for blood transfusion, prolonged hospital stay, ICU admission, and may sometimes require even hysterectomy. Accurate diagnosis requires accurate scanning of all anterior and low lying gestational sacs in a post-cesaraen pregnancy. Transvaginal sonography is the imaging modality of choice. Empty uterine cavity with clearly visualized endometrium; empty cervical canal; gestational sac in the anterior LUS; and absent or deficient intervening myometrium between the gestational sac and bladder wall gives diagnostic clues of CSP. MRI can resolve any doubtful USG findings but is rarely required. Timely surgical intervention in emergency can prevent hysterectomy. Medical management of stable CSP is feasible with systemic methotrexate therapy and follow up. It is critical to accurately localize early pregnancy in post cesarean gestations to recognize ectopic sac at the scar and manage CSP successfully.

## Conclusion

Diagnosis of CSP requires an accurate and early transvaginal scan of all anterior and low lying gestational sacs in a post-cesaraen pregnancy. The management of CSP often requires multi-modality treatment for successful outcome.
